# The unique face of comorbid anxiety and depression: increased interoceptive fearfulness and reactivity

**DOI:** 10.3389/fnbeh.2022.1083357

**Published:** 2023-01-23

**Authors:** Maria Ironside, Danielle C. DeVille, Rayus Tiberius Kuplicki, Kai Ping Burrows, Ryan Smith, Adam R. Teed, Martin P. Paulus, Sahib S. Khalsa

**Affiliations:** ^1^Laureate Institute for Brain Research, Tulsa, OK, United States; ^2^Oxley College of Health Sciences, University of Tulsa, Tulsa, OK, United States; ^3^Department of Psychiatry, University of California, San Diego, La Jolla, CA, United States

**Keywords:** anxiety, depression, fear, threat, interoception, nociception, pain, interoceptive awareness

## Abstract

Anxiety and depression commonly co-occur, yet the underlying brain and behavioral processes are poorly understood. Here we examined the hypothesis that individuals with comorbid anxiety and depression would show increased fearful reactivity to an aversive interoceptive perturbation relative to depressed-only individuals. One-hundred and eighty anxious and/or depressed participants from the Tulsa 1000 study completed multi-level behavioral or functional magnetic resonance imaging assessments of interoception and nociception including breath-hold and cold-pressor challenges, and heartbeat perception and interoceptive attention tasks. One-hundred and four individuals with comorbid depression and anxiety disorders (Dep+Anx) were propensity matched with 52 individuals with depression-only (Dep). Data were analyzed using mixed-effects linear regression. The Dep+Anx group showed significantly greater self-reported fear of suffocation during breath holding (Wilcoxon *r* = 0.23) and reduced cold pain tolerance (*R*^2^ = 0.027) signified by hand removal during immersion. However, these groups did not differ with respect to neutrally-valenced behavioral indices of heartbeat perception or neural indices of interoceptive attention. Individuals with comorbid depression and anxiety, vs. those with only depression, show increased respiratory fearfulness and nociceptive reactivity during perturbations of these signals, whilst showing similar interoceptive awareness in the absence of perturbation. Our findings suggest that individuals with comorbid anxiety and depression process aversive interoceptive and nociceptive signals differently than those with depression alone, providing support for a process model of increased threat sensitivity and hyperarousal in anxious depression.

## Introduction

Nearly half of all individuals with major depressive disorder (MDD) also have an anxiety disorder (Kessler et al., 2015). Thus, comorbid MDD and anxiety disorders (e.g., generalized anxiety disorder, social anxiety disorder, panic disorder, or specific phobia) are among the most common presentations of affective psychopathology in psychiatric settings. Anxious depression is associated with greater treatment resistance (Ionescu et al., 2014), worse side effect profiles (Gaspersz et al., 2017), poorer treatment outcomes (Penninx et al., 2011), quicker symptom relapse (Fava et al., 2008) and higher levels of suicidal ideation (Kessler et al., 2015) than non-anxious depression. However, treatment engagement appears to be higher in this comorbid group (Kessler et al., 2015). Despite the pervasiveness of anxious depression, the neural and behavioral processes characterizing this form of psychopathology remain incompletely understood. A better understanding of the underlying process dysfunctions may be an important first step in the development of more mechanistically oriented interventions.

Depression and anxiety share abnormalities across a number of different neurocognitive processes, such as negative affect (Clark and Watson, 1991), life stress (Syed and Nemeroff, 2017), and cognitive function (Millan et al., 2012). However, studies comparing the mechanisms of anxious vs. non-anxious depression are rare, which may be one factor impeding improvements in treatment development. Hyperarousal, defined as an abnormal state of increased responsiveness to stimuli that is marked by various physiological and psychological symptoms, is a feature of both anxiety and anxious depression (Clark and Watson, 1991) that is not present in non-anxious depression. It may therefore be a useful intermediary target mechanism. Since anxiety disorders are also commonly characterized by abnormalities in the domain of interoception (Khalsa et al., 2018), the mechanistic probing of interoception in anxious depression might inform a deeper understanding of the underlying pathophysiology that could help to tease apart the differences between anxious and non-anxious depression.

Interoception refers to the process by which the nervous system senses, interprets, and integrates signals originating from within the body (Khalsa et al., 2018). Interoceptive awareness has many features, including: (1) interoceptive attention (i.e., cued or spontaneous focus on internal physiological sensations); (2) interoceptive accuracy (i.e., the match between true and consciously perceived internal physiological sensations); (3) interoceptive magnitude (i.e., the perceived intensity of internal physiological sensations (Khalsa and Lapidus, 2016; Khalsa et al., 2018); (4) interoceptive sensibility (i.e., self-perceived tendency to focus on interoceptive stimuli (Garfinkel et al., 2015); and (5) interoceptive insight (i.e., the metacognitive correspondence between self-perceived and objective measures of interoceptive accuracy). Whether, and how often interoceptive processes enter awareness varies according to the organ system and level of physiological arousal, and therefore multimodal assessments involving self-report, behavioral, and physiological forms of measurement across multiple domains may be important for gaining an informed perspective on this process. The insular cortex is a key hub within the neural circuitry implicated in interoception (Berntson and Khalsa, 2021; Nord et al., 2021). Related to this, models of embodied emotion (Craig, 2008; Smith and Lane, 2015; Barrett, 2017) have been proposed that integrate interoception with wider emotional processing and motivation. In this view, the co-activation of regions such as the anterior insula and anterior cingulate cortex (ACC) during emotion processing (Thielscher and Pessoa, 2007) and across psychiatric disorders (McTeague et al., 2020) is consistent with the view that an emotional experience involves body states detected *via* interoceptive processes that may be altered in psychopathology. It follows from this that functional neuroimaging tasks which specifically perturb interoception can elucidate the neural circuits underlying interoceptive psychopathology (Smith et al., 2021; Teed et al., 2022). Furthermore, the anatomical overlap between circuits associated with fear conditioning (Maren and Quirk, 2004) and the central autonomic network (Thayer and Lane, 2000; Smith et al., 2017) includes the ventromedial prefrontal cortex (vmPFC), cingulate cortex, insula, and amygdala. This overlap forms the basis of a neurovisceral model of fear conditioning (Battaglia and Thayer, 2022) and makes the study of aversive interoception particularly relevant.

Aberrant interoception has been strongly linked to many forms of psychopathology beyond anxious depression, including MDD (Avery et al., 2014; Wiebking et al., 2015), substance use disorders (Paulus and Stewart, 2014; Stewart et al., 2020), eating disorders (Khalsa et al., 2015; Kerr et al., 2016; Berner et al., 2018), suicidal behaviors (DeVille et al., 2020, 2021), and schizophrenia (Yao and Thakkar, 2022). Yet findings in depression are mixed. For example, in one study (Dunn et al., 2007), a severely depressed group did not differ from healthy comparators in heartbeat perception accuracy, whereas a more moderate group showed significantly lower accuracy, as also seen in other studies (Terhaar et al., 2012). This could suggest a non-linear effect of depression on interoception (Eggart et al., 2019). Alternatively, it might be explained by the heterogeneity of depression and/or the inclusion of co-morbid anxiety in the patient groups under investigation. Depression has been generally associated with blunted cardiac interoceptive awareness (Pollatos et al., 2009; Furman et al., 2013), whereas anxiety has often been conceptually associated with increased sensitivity (Van der Does et al., 2000; Domschke et al., 2010), perhaps driven by abnormally heightened interoceptive prediction signals of a somatic threat (Paulus and Stein, 2006; Khalsa and Feinstein, 2018). Interactions in the effect of depressive and anxiety symptoms on interoception have been suggested dimensionally in healthy individuals (Pollatos et al., 2009) and those with mood disorder symptoms (Dunn et al., 2010), although firm conclusions from such studies are limited by reliance on a measure of cardiac interoception with several drawbacks (Desmedt et al., 2018; Ring and Brener, 2018; Corneille et al., 2020; Ferentzi et al., 2022). Despite the available evidence of transdiagnostic interoceptive dysfunction, there is little work comparing interoception in individuals with MDD vs. those with MDD and comorbid anxiety.

The goal of the current cross-sectional investigation was to determine whether individuals with anxious depression show different sensitivity to interoceptive sensations of a threat compared to non-anxious depressed individuals. Our approach was to compare a sample of depressed individuals who were propensity-matched based on demographic characteristics to individuals with comorbid anxiety and depression using an interoceptive battery of tasks developed to measure different aspects of cardiorespiratory interoceptive and nociceptive processing. Our overarching hypothesis was that individuals with anxious depression would show increased interoceptive reactivity to aversive sensations relative to their depressed counterparts.

## Materials and methods

### Participants

Data analyzed for the current study included a group of 180 anxious and/or depressed participants (132 female) who were selected from the first 500 participant cohort of the Tulsa 1000 project (Victor et al., 2018), a naturalistic longitudinal study that recruited a community sample of 1,000 individuals with various forms of psychopathology, based on the dimensional NIMH Research Domain Criteria framework. Participants were between 18 and 55 years of age at the time of assessments (mean age = 35.6, standard deviation = 11.5). Depression and anxiety screening inclusion criteria included the following symptom scores: (1) Patient Health Questionnaire [PHQ-9, (Levis et al., 2019)] ≥10; and/or (2) Overall Anxiety Symptom and Impairment Scale [OASIS, (Campbell-Sills et al., 2009)] ≥8. Exclusion criteria were positive urine drug screen; lifetime bipolar, schizophrenia spectrum, antisocial personality, or obsessive compulsive disorders; current suicidal ideation with intent or plan; moderate to severe traumatic brain injury; severe and or unstable medical concerns; changes in psychiatric medication dose in the last 6 weeks; and Magnetic Resonance Imaging (MRI) contraindications. Participants who met the criteria for substance use or eating disorders were excluded from analysis in the current study. Full inclusion/exclusion criteria can be found in the supplement and the parent project protocol article (Victor et al., 2018). Ethical approval was obtained from the Western Institutional Review Board. All participants provided written informed consent prior to participation, in accordance with the Declaration of Helsinki, and received financial compensation for participation. ClinicalTrials.gov identifier: #NCT02450240.

The initial analysis included 52 participants with non-anxious depression (Dep) and 128 participants with comorbid depression and anxiety disorder (Dep+Anx). To reduce potential confounds, 104 participants from the Dep+Anx group were propensity matched for age, sex, and education at a ratio of 2:1 with 52 participants from the Dep group using the MatchIt package in R (Ho et al., 2011). These two groups did not differ in their level of depression (PHQ-9), age, or body mass index (see Table 1) but, crucially, had significantly different levels of self-report anxiety sensitivity [Anxiety Sensitivity Index; ASI, (Taylor et al., 2007)] and anxiety severity (OASIS). There were significantly more racially white people in the Dep+Anx group but other racial groups did not significantly differ.

**Table 1:** Demographics of propensity matched Depressed (Dep) and depressed and anxious (Dep+Anx) participants.

**Measure**	**Dep**		**Dep+Anx**		**Significance**
**N**	52		104		
**Age (M, SD)**	37.8	11.9	36.2	11	*t*_(89)_ 1.616, *p* = 0.11
**Female (N, %)**	36	69%	75	72%	*χ*^2^_(1)_ 0.629, *p* = 0.43
**Race: Asian**	2	4%	1	1%	*χ*^2^_(1)_ 2.12, *p* = 0.15
**Race: Black**	6	11%	7	7%	*χ*^2^_(1)_ 2.033, *p* = 0.15
**Race: White**	42	80%	93	89%	***χ*^2^_(1)_ 4.059, *p* = 0.04**
**Race: Native American or Pacific Islander**	5	10%	21	20%	*χ*^2^_(1)_ 2.283, *p* = 0.13
**Latinx ethnicity (N, %)**	1	2%	5	5%	*χ*^2^_(1)_ 0.426, *p* = 0.51
**Body Mass Index (M, SD)**	28.7	5.3	28.7	5.6	*t*_(97)_ 0.009, *p* = 0.99
**Education (some college or higher; N, %)**	45	87%	86	82%	*t*_(127)_ 1.311, *p* = 0.19
**Depression (PHQ-9; M, SD)**	13.48	4.08	12.72	4.91	*t*_(113)_ 1.069, *p* = 0.29
**Anxiety sensitivity (ASI) (M, SD)**	19.8	10.9	27.7	14	***t*_(122)_ 4.606, *p* < 0.001**
**Anxiety (OASIS; M, SD)**	8	3.6	10.2	3.3	***t*_(84)_ 4.021, *p* < 0.001**
**Psychiatric medication**	31	60%	72	69%	*χ*^2^_(1)_ 1.920, *p* = 0.17

M, mean; SD, standard deviation; N, number; %, percentage; PHQ-9, Patient Health Questionnaire 9-item version; ASI, Anxiety Sensitivity Index; OASIS, Overall Anxiety Severity and Impairment Scale. Bolded values represent a significant difference between the groups at *p* <= 0.05.

### General procedure

General procedures included a clinical interview session, a neuroimaging session, and a behavioral (psychophysiology) session—all completed within 2 weeks on average. Although the parent project (i.e., the Tulsa 1000) consisted of a broader range of protocols (comprising ~23.5 h of testing), only details relevant to the current study (comprising ~7 h of testing) are presented here. See the supplement and protocol article (Victor et al., 2018) for details.

Study clinicians (masters or nurse level assistants, supervised by licensed clinical psychologists and board-certified psychiatrists) administered the MINI (Sheehan et al., 1998) structured clinical interview to establish the current presence of depressive episodes, anxiety disorder, substance use disorder or eating disorders and to rule out bipolar disorder, schizophrenia, personality disorders or obsessive compulsive disorder. During this session, participants also provided self-reported information on sociodemographics (i.e., age, education, income, ethnicity, and race), anxiety [OASIS, Anxiety Sensitivity Index; ASI (Taylor et al., 2007)], approach and avoidance motivation [Behavioral Inhibition/Behavioral Approach Scale; BIS/BAS (Carver and White, 1994)], depression (PHQ-9), emotional processing [Positive and Negative Affect Schedule; PANAS (Watson et al., 1988)], impulsivity [UPPS Impulse Behavior Scale (Whiteside et al., 2005)], pleasure [Temporal Experience of Pleasure Scale; TEPS (Gard et al., 2006)], substance use (Patient Reported Outcome Measurement Information System; PROMIS), and trauma [Traumatic Events Questionnaire (Vrana and Lauterbach, 1994)]. For the current study, we focused on dimensional measures of anxiety/threat sensitivity (ASI), and depression [PROMIS depression scale (Cella et al., 2010)].

### Interoceptive battery

The interoceptive battery of tasks consisted of all interoceptive tasks included in the T1000 study and are described fully in DeVille et al. (2020, 2021) and Lapidus et al. (2020). Brief descriptions follow below.

### Breath-hold challenge

Each participant completed two inspiratory breath-hold trials (Asmundson and Stein, 1994), providing a brief measure of endogenous sensitivity to respiratory perturbation. Participants were seated in front of a computer screen, fitted with a respiration belt and electrocardiogram sensors (Biopac Systems, Inc.), and provided with a nose clip to prevent inadvertent respirations (see Lapidus et al., 2020 for a detailed description of the procedure). During normal breathing, concentrations of oxygen and carbon dioxide were analyzed from their exhaled air, providing a baseline measurement. Participants were then instructed to inhale maximally and, at the end of inhalation, to begin holding their breath for as long as they were able to tolerate. Participants were instructed to exhale into a capnometer-connected breathing tube (Oxigraf, Inc.) when they were no longer able to tolerate the breath hold. Following each breath hold, participants provided subjective ratings of the task (i.e., intensity, unpleasantness, and difficulty) as well as ratings of associated psychological experiences (i.e., stress, required effort, breathlessness, urge to breathe, sensations of suffocation, fear of suffocation) on visual analog scales (VAS) ranging from 0 (not at all) to 100 (extremely). We hypothesized that the Dep+Anx group would report more negative ratings/experiences of the breath-hold than the Dep group.

### Cold-pressor challenge

Participants immersed their dominant hand in a circulating pool of water cooled to 6 degrees Celsius (Thermo Fisher Scientific Inc., Arctic A25 Circulator). They were asked to keep their hand submerged for as long as they could tolerate. During the task, participants made continuous real-time pain intensity ratings on a scale ranging from 0 (no pain) to 100 (worst pain imaginable). These ratings were used to calculate each participant’s peak pain rating, as well as the time to reach ratings of mild (25/100), moderate (50/100), and peak pain. Afterward, each participant provided VAS ratings of unpleasantness, difficulty, and stress ranging from 0 (not at all) to 100 (extremely). See DeVille et al. (2020) for further details. We hypothesized that the Dep+Anx group would remove their hand more quickly than the Dep group and rate the experience more negatively.

### Heartbeat-perception task

To assess cardiac interoception, participants performed a heartbeat-tapping task across three interoceptive conditions. During each condition, participants were instructed to press a key on a keyboard every time they felt their heartbeat, without taking their pulse. In the first trial (“guess”), subjects were instructed to tap every time they felt their heartbeat without taking their pulse. Guessing was encouraged if they felt unsure. In the next trial (“no guess”), guessing was discouraged, and participants were asked to tap only when they felt confident in feeling their heartbeat. In the final trial (“breath hold perturbation”), participants were instructed to inhale deeply, hold their breath, and tap along with their perceived heartbeats while sustaining the breath hold, without guessing. The breath-hold was expected to amplify cardiac sensations and presumably increase heartbeat-perception accuracy, and computational work in this task has shown a failure to adapt interoceptive processing in psychopathology (Smith et al., 2020). Afterward, participants provided VAS ratings ranging from 0 (not at all) to 100 (extremely) to indicate their perceived heartbeat intensity, confidence in their ability to accurately estimate their heartbeat, and their assessment of task-related difficulty. Interoceptive accuracy was quantified using the Beat-to-Tap consistency metric. This measure is described fully by Smith et al. (2021). Briefly, it quantifies how consistent the delay is between when a person taps and the event of interest (heartbeat or tone), compared to what would be expected if they tapped randomly (i.e., if taps did not track the timing of heartbeats or tones, irrespective of potential reaction time differences). This was regarded as an exploratory analysis to assess potential differences in the consistency of interoceptive estimates in non-threatening settings.

### Interoceptive attention task

Participants completed two runs of the visceral interoceptive attention (VIA) task (Simmons et al., 2013), wherein they were presented with three conditions cued by a visually presented word (10 s duration): (1) “heart” cued internal attention toward heartbeat sensations; (2) “stomach” cued internal attention toward stomach sensations; and (3) “target” cued external attention toward word color changes at varying intensities. The VIA task has been previously shown to be effective at mapping the neural signal associated with interoceptive attention in several psychiatric and non-psychiatric populations (Kerr et al., 2016; DeVille et al., 2020; Stewart et al., 2020). Participants were asked to provide ratings of stimulus intensity (0 = “no sensation” to 6 = “extreme sensation”) after 50% of trials, which also helped to ensure they remained awake and were attending to the task. Each run included six trials per condition (intertrial interval range 2.5–12.5 s). Regions of interest (ROIs) from within the insula were chosen as these are typical regions evoked by this task (see above citations). The vmPFC was also examined following our recent findings showing reduced vmPFC activation was related to differences in interoception between individuals with generalized anxiety disorder compared to healthy comparisons (Teed et al., 2022). This was also regarded as an exploratory analysis to assess any differences in neural signals underlying interoceptive attention in non-threatening settings.

### Magnetic resonance imaging (MRI) data acquisition and preprocessing

Structural and functional MRI data were acquired on two identical GE Discovery MR750 3T scanners operating identical pulse sequences for functional [repetition time (TR)/echo time (TE) = 2,000/27 ms, the field of view (FOV)/slice = 240/2.9 mm, 128 × 128 matrix, 39 axial slices, 180 TRs] and structural scans [magnetization prepared rapid acquisition gradient recalled echo (MP-RAGE) TR/TE = 5/2.012 ms, FOV/slice = 240×192/0.9 mm, 186 axial slices]. Single-subject preprocessing was completed using Analysis of Functional NeuroImages (AFNI) software (Cox, 1996). The first three TRs were discarded, followed by despiking, slice-timing correction, co-registration to anatomical volumes, motion correction, transformation to Montreal Neurological Institute space *via* an affine transformation, application of a 4 mm Gaussian full-width at half-max smoothing kernel, and a voxelwise general linear model analysis. Block regressors were convolved with a canonical hemodynamic response function and used to model blood oxygen level dependent (BOLD) response for heart, stomach, and target conditions. Six motion parameters (three translations and three rotations) were included as nuisance regressors. Censoring was done at the regression step by removing volumes with either a Euclidean norm of the derivatives of the six motion parameters greater than 0.3 mm or greater than 10% outlier voxels, determined by 3dToutcount. Percent signal change during each condition was defined as the estimated beta coefficient from single-subject analysis, which was relative to the implicit baseline during unmodeled fixation and scaled by the zeroth order regressor to convert to percent signal change.

### Data analysis

For analyses with repeated measures, linear mixed effects (LME) regression analyses were conducted using the lmerTest package in R. For analyses without repeated measures, data were analyzed using linear models in R. Analysis of variance (ANOVA) was applied to each regression model to examine *F* tests for interactions and main effects. Significant interactions were followed up with an examination of fixed-effects. The Kenward–Roger approximation of degrees of freedom was used. Effect sizes (*R*^2^) for significant findings were estimated using the r2glmm package in R (Jaeger, 2017). Benjamini–Hochberg adjustments were applied within each set of analyses to account for repeated testing. The ggplot2 package (Wickham et al., 2016) was used to visualize data.

There were some missing data from certain tasks on the basis of nonadherence to task instructions (i.e., manually taking their pulse during the heartbeat-perception task; 19 participants), experimenter error (three participants) and refusal to complete the cold-pressor task, (one participant). Data for these individuals were excluded.

#### Analysis of behavioral tasks

For the breath-hold challenge, cold-pressor challenge, and heartbeat-tapping task, LME models were used to examine the relationship between the group and the dependent measure for each task. A random effect of participants (random intercept) was included within each model and covariates of Age, Sex, and Medication status were included. Individual fixed-effects specifications are provided for each model below. VAS ratings for each task were also compared between groups. Since a proportion of the VAS ratings were not normally distributed, Mann-Whitney tests, which are robust to deviations from normality, were used to compare ratings between groups, and a Wilcoxon R was reported for effect size.

#### Breath-hold and cold-pressor challenges

For the breath-hold task, duration in seconds was the primary dependent measure. Group, trial repetition, and the interaction between group and trial were included as fixed effects. For the cold pressor challenge, duration in seconds was the primary dependent measure. Group, time point (i.e., markers of mild pain, moderate pain, peak pain, and task discontinuation), and the interaction between group and time point were included as fixed effects.

#### Heartbeat-perception task

To counteract known limitations of the traditional formula used to measure heartbeat accuracy, we used our previously developed beat-to-tap consistency measure (Smith et al., 2021). We focused on the no-guess and perturbation conditions of this task due to the known limitations of the guessing condition (Desmedt et al., 2018). Group, condition (i.e., no-guess and perturbation), and the interaction between group and condition were included as fixed effects.

#### Interoceptive attention task

ROI analyses were performed focusing on insular and vmPFC subregions. Specifically, we focused on group differences in signal change in BOLD on the contrast of interoceptive vs. exteroceptive attention (INT-EXT) within six bilateral insula ROIs based on Brainntome atlas (Fan et al., 2016) subregions (hypergranular, ventral agranular, dorsal agranular, ventral dysgranular/granular, dorsal granular and dorsal dysgranular insula), and four vmPFC ROIs based on meta-analyses of fMRI studies related to valence processing (Lindquist et al., 2016), sympathetic autonomic control (Beissner et al., 2013) and self-processing (Murray et al., 2012). Group, laterality, and the interaction between group and laterality were included as fixed effects. An exploratory whole brain analysis was also carried out in AFNI using 3dMVM on the contrast of interoceptive vs. exteroceptive attention (INT-EXT), with an initial voxel height threshold of *p* < 0.001 and a cluster extent threshold of 20 voxels.

#### Power calculation

Power was calculated using G-Power software. Assuming small effect sizes (Cohen *f* = 0.1/Cohen *d* = 0.2), a critical alpha of *p* = 0.05, and the given sample size, the analyses had the following power to detect an effect: Cold-Pressor Challenge = 90%; Breath-Hold Challenge and Heartbeat-Perception Task = 76%; VIA Task = 33%.

## Results

### Breath-hold challenge

**Figure 1:**
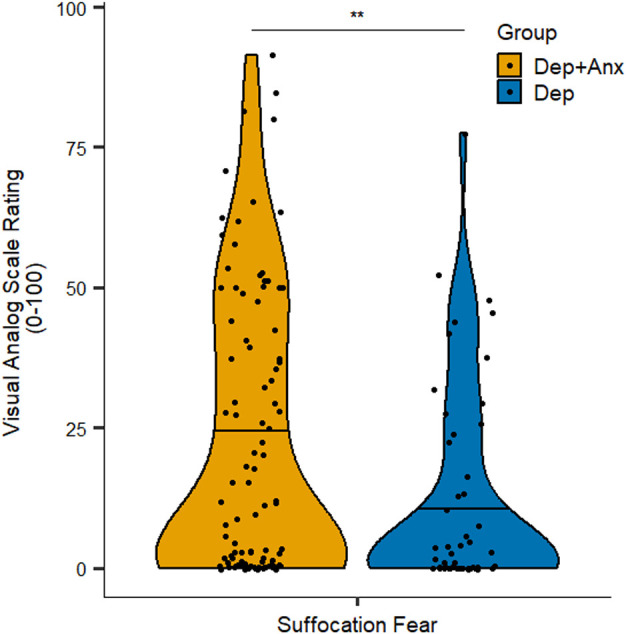
Self-report ratings of suffocation fear in the Breath-Hold Task. Note that the Dep+Anx group reported significantly higher suffocation fear than the Dep group. Violin plots visualize distribution of the data, horizontal line represents the median. Dep+Anx, comorbid depression and anxiety disorders; Dep, depression only. **p < 0.01.

Anxious depression was significantly associated with VAS ratings of fear of suffocation, such that the Dep+Anx group reported greater fear of suffocation [mean (*M*) = 22.5, standard deviation (SD) = 25.0] than those in the Dep group (*M* = 12.0, SD = 18.2; *p* = 0.005, corrected *p* = 0.046, Wilcoxon *r* = 0.23; see Figure 1). Feelings of suffocation were also greater in the Dep+Anx group (*M* = 43.2, SD = 29.3) compared to the Dep group (*M* = 32.8, SD = 28.1), but this did not survive correction for multiple comparisons (*p* = 0.03, corrected *p* = 0.136, Wilcoxon *r* = 0.18). All other group comparisons of VAS ratings were nonsignificant (corrected *p* ranging from 0.583 to 0.821, see Table 2 for full details). For breath-hold duration, there was a significant main effect of trial, *F*_(1,154)_ = 31.61, *p* < 0.001, *R*^2^ = 0.17, indicating that both groups held their breath for significantly longer in the second trial compared to the first trial. However, there was no significant effect of *group*, *F*_(1,77)_ = 1.46, *p* = 0.22, and no *Group × Trial* interaction, *F*_(1,154)_ = 0.56, *p* = 0.45.

**Table 2:** Full statistical results from Breath-hold Challenge, Heartbeat-Perception Task, Cold Pressor Challenge, and Interoceptive Attention Task.

**Measure**	**Depressed only**		**Depressed + Anxious**		**Significance (uncorrected)**
***Breath-Hold Challenge***	**mean**	**SD**	**mean**	**SD**	
Duration*	54.38	25.73	50.04	25.41	*F*_(1,177)_ = 1.46, *p* = 0.22
O_2_*	12.51	1.88	12.90	2.02	*F*_(1,174)_ = 2.18, *p* = 0.14
CO_2_*	5.92	0.80	5.81	0.70	*F*_(1,152)_ = 2.14, *p* = 0.15
VAS—Effort	80.85	17.55	81.14	13.07	*W* = 2,438, *p* = 0.73
VAS—unpleasantness	73.56	18.44	70.48	22.20	*W* = 2,395, *p* = 0.61
VAS—intensity	70.49	21.78	69.83	18.83	*W* = 2,406, *p* = 0.64
VAS—difficulty	54.28	23.40	58.09	24.09	*W* = 2,781, *p* = 0.26
VAS—stress	43.86	23.63	49.27	26.75	*W* = 2,766, *p* = 0.20
VAS—breathless	63.86	22.87	62.31	23.87	*W* = 2,417, *p* = 0.89
VAS—urge	73.32	18.48	71.22	21.41	*W* = 2,361, *p* = 0.72
VAS—feelings of suffocation	32.82	28.11	43.23	29.31	***W* = 2,985, *p* = 0.03**
VAS—suffocation fear	11.97	18.20	22.51	25.04	***W* = 3,134, *p* = 0.005**
*Heartbeat-Perception Task*					
Beat-to-tap consistency	0.49	1.59	0.60	1.86	*F*_(1,238)_ = 1.26, *p* = 0.26
VAS—confidence	45.43	24.74	45.83	24.35	*W* = 2,650, *p* = 0.99
VAS—difficulty	64.04	23.73	59.68	26.29	*W* = 2,444, *p* = 0.43
VAS—intensity	33.81	25.11	31.00	24.24	*W* = 2,465, *p* = 0.48
*Cold-Pressor Challenge*					
Sec to mild pain	9.53	7.13	10.15	8.03	*t*_(449)_ = 0.14, *p* = 0.88
Sec to moderate pain	18.61	17.59	17.03	12.49	*t*_(449)_ = 0.36, *p* = 0.72
Sec to peak pain	42.03	24.54	37.71	26.65	*t*_(449)_ = 0.96, *p* = 0.32
Sec to removal	75.25	44.24	59.51	39.28	***t*_(449)_ = 3.62, *p* = 0.0003**
VAS—pain	81.74	14.21	81.61	14.37	*W* = 2,728, *p* = 0.93
VAS—unpleasantness	86.87	15.60	88.64	14.61	*W* = 2,919, *p* = 0.42
VAS—difficulty	71.85	27.54	74.63	28.89	*W* = 2,911, *p* = 0.44
VAS—stress	58.69	31.87	64.75	27.65	*W* = 2,965, *p* = 0.33
*Interoceptive Attention Task*					
*Contrast of INT—EXT*					
Hypergranular insula	0.0010	0.0011	0.0010	0.0013	*F*_(1,140)_ = 0.02, *p* = 0.89
Dorsal granular insula	0.0005	0.0010	0.0004	0.0012	*F*_(1,140)_ = 0.07, *p* = 0.79
Dorsal dysgranular insula	0.0011	0.0013	0.0011	0.0015	*F*_(1,140)_ = 0.001, *p* = 0.97
Ventral agranular insula	−0.0001	0.0013	−0.0002	0.0016	*F*_(1,140)_ = 0.05, *p* = 0.83
Dorsal agranular insula	−0.0009	0.0016	−0.0009	0.0017	*F*_(1,140)_ = 0.01, *p* = 0.92
Ventral dysgranular insula	0.0009	0.0014	0.0007	0.0014	*F*_(1,140)_ = 0.67, *p* = 0.41
vmPFC (self-related processing**)	0.59	10.81	2.83	13.66	*F*_(1, 140)_ = 0.99, *p* = 0.32
vmPFC (valence processing**)	3.66	6.46	2.54	7.71	*F*_(1,140)_ = 0.75, *p* = 0.39
vmPFC 3 (sympathetic processing**)	3.49	7.81	1.75	9.28	*F*_(1,140)_ = 1.27, *p* = 0.27
vmPFC 4 (sympathetic processing**)	1.79	7.55	1.38	10.40	*F*_(1,140)_ = 0.06, *p* = 0.81

M, mean; SD, standard deviation; VAS, Visual Analogue Scale. *Values averaged across two runs as there was no Group × Timepoint interaction. **vmPFC ROIs from Teed et al. (2022). Bolded values represent a significant difference between the groups at *p* <= 0.05.

### Heartbeat-perception task

For heartbeat beat-to-tap consistency (see Supplementary Material), mixed-effects linear regression showed that there were no significant effects of *group*, *F*_(1,238)_ = 1.26, *p* = 0.26, and the interaction between *group × condition* was also not significant, *F*_(1,126)_ = 2.14, *p* = 0.15. Similarly, Wilcoxon rank sum tests indicated that there were no group differences in VAS ratings of overall difficulty, intensity, or confidence regarding the heartbeat-perception task (corrected *p* ranging from 0.716 to 0.992). See Table 2 for full details.

### Cold-pressor challenge

**Figure 2:**
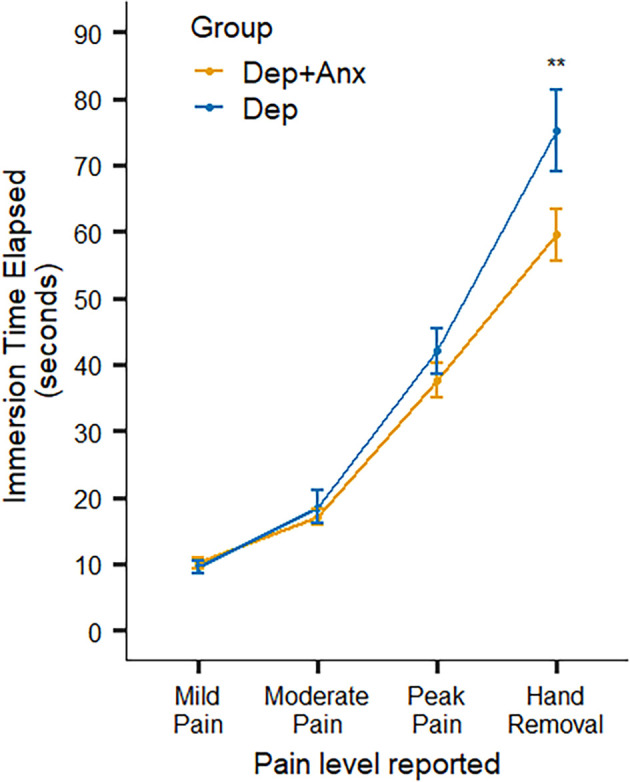
Self-report ratings of pain and behavioral hand removal in the Cold-Pressor Task. Note that the Dep+Anx group removed their hand significantly more quickly than the Dep group. Error bars represent ± 1 SE of the mean. Dep+Anx, comorbid depression and anxiety disorders; Dep, depression only. **p < 0.01.

Across all participants, mixed-effects linear regression found that the cold-pressor challenge elicited increased pain ratings over time (*F*_(3,462)_ = 121.46, *p* < 0.001, *R*^2^ = 0.585). There was a significant interaction between *timepoint × group* (*F*_(3,462)_ = 4.32, *p* = 0.005, *R*^2^ = 0.027). This was driven by the Dep group keeping their hands submerged in the cold water for significantly longer (approximately 16 s longer) than the Dep+Anx group after reaching their peak pain level (*t*_(449)_ = 3.62, *p* = 0.0003, *b* = 15.73), without any statistically significant differences in the amount of time taken to reach self-reported mild, moderate, and peak pain levels (Figure 2 and Table 2). We report fixed effects and model summary values in Table 3. Wilcoxon rank sum tests indicated that there were no significant differences in average ratings of unpleasantness, pain, difficulty, and stress between the two groups (corrected *p* ranging from 0.581 to 0.930).

**Table 3:** Model parameters from the LME model for the Cold-Pressor Challenge.

	**value**				
***Predictors***	***std. Beta***	***std SE***	***std. 95% CI***	***Statistic***	***p***
**(Intercept)**	−0.68	0.08	−0.83 to −0.53	4.04	**<0.001**
**timepoint [moderate]**	0.21	0.09	0.04–0.37	2.41	**0.016**
**timepoint [peak]**	0.82	0.09	0.66–0.99	9.64	**<0.001**
**timepoint [removal]**	1.48	0.09	1.31–1.64	17.28	**<0.001**
**group [Dep+Anx]**	−0.02	0.13	−0.27–0.24	−0.14	0.888
timepoint [moderate] * group [Dep+Anx]	0.07	0.15	−0.22–0.36	0.44	0.659
timepoint [peak] * group [Dep+Anx]	0.15	0.15	−0.14–0.44	1	0.318
timepoint [removal] * group [Dep+Anx]	0.49	0.15	0.20–0.78	3.3	**0.001**
**Random Effects**					
**σ^2^**	424.66				
**τ_00 id_**	230.68				
**ICC**	0.35				
**N_id_**	156				
**Observations**	624				
**Marginal *R*^2^, Conditional *R*^2^**	0.418, 0.623				

Note: std., standardized; SE, standard error; CI, confidence interval. σ^2^, variance of residual; τ_00 id_, variance of random effect of subject (intercept); ICC, intraclass correlation coefficient; N_id_, number of subjects. Bolded values represent a significant difference between the groups at *p* <= 0.05.

### Interoceptive attention task

For the exploratory insula and vmPFC ROI analyses, mixed effects regression analyses indicated that there were no significant effects of *group* (all corrected *p*’s > 0.41, see Table 2) or *group* × *laterality* interactions (all corrected *p*’s > 0.552) for any of the six insula or four vmPFC ROIs. A subsequent exploratory whole brain analysis did not reveal any significant clusters of interest differentiating the groups. However, across both the groups there was clear evidence of dorsal mid-insula activation to the contrast of INT-EXT attention (all corrected *p*’s < 0.001), consistent with prior studies.

## Discussion

This study examined whether individuals with anxious depression exhibit increased sensitivity to aversive interoceptive signals relative to demographically matched individuals with non-anxious depression. There were two main results: first, the Dep+Anx group reported greater fear of suffocation during a breath-hold task, despite similar breath-hold durations compared to the Dep group. Second, the Dep+Anx group removed their hand from painful cold water more quickly than the Dep group, despite having similar self-reported pain ratings over time. However, there were no differences between the groups on the heartbeat-perception task or the VIA task. This could be explained by the Dep+Anx group (relative to Dep only and possibly relative to healthy comparisons) perceiving body signals that accompany the breath-hold task as more threatening and reporting this subjectively. Then, in the cold-pressor task this Dep+Anx group reacted in such a way as to avoid threatening nociceptive signals by terminating the trial earlier. Together, these results support the hypothesis that anxious depression is characterized by an exaggerated response to aversive interoceptive signals relative to those with non-anxious depression and reduced tolerance of nociceptive threat. To our knowledge, this is the first time that differences in interoceptive reactivity between anxious vs. non-anxious depression have been identified, which was specifically enabled by our propensity-matching approach.

Previously, we showed increased emotional reactivity to threat in a startle EMG task in anxious depression compared to non-anxious depression, using the same propensity-matched sample (Ironside et al., 2022). An association between emotional reactivity and increased interoceptive awareness has also been previously shown (Pollatos et al., 2007; McTeague et al., 2011), which may suggest that a chronically increased outflow of sympathetic signals might be one variable contributing to the establishment of high interoceptive sensitivity/fearfulness, though potentially to the detriment of interoceptive accuracy (Paulus and Stein, 2010). Interestingly, in the cold pressor challenge, both groups had similar pain ratings over time, but the Dep+Anx group turned these feelings (pain) into action (hand removal) more quickly than the Dep group. This could be explained by reduced cognitive control or stronger emotional experiences in anxious depression for the same level of pain. Another possibility is a potential limbic-motor dysfunction in non-anxious depression, which prevents the interoceptive signal from activating motivational resources. In contrast, we see at least a preservation, if not an exaggeration of motivational activation to bodily sensations of threat in anxious depression. One explanation of altered interoception in psychiatric illnesses suggests a combination of rigid and exaggerated expectations of aversive interoceptive experiences and low signal-to-noise ratio of visceral input to the insula, resulting in erroneous evaluation of benign signal changes as significant, with accordingly motivated action (Paulus et al., 2019). It is unclear if the group difference seen here represents limbic motor dysfunction in non-anxious depression or erroneous evaluation in anxious depression, or (likely) a combination of the two.

Previous work based on symptom patterns supports a tripartite model (Clark and Watson, 1991), i.e., a bifactor model consisting of a general negative affect factor and specific depression and anxiety factors. The anxiety-specific factor in the tripartite model is hyperarousal. In the breath-hold and cold pressor tasks, the aversive interoceptive signal activates a defensive system. As expected from the tripartite model, the Dep+Anx group show hyperarousal, represented by higher fear of suffocation and quicker hand removal, which is not observed in the Dep group, suggesting a key difference between these sub-groups. In terms of behavior, the approach-withdrawal model helps tease apart the different factors present in anxious depression, with depression being associated with reduced approach and anxiety being associated with increased avoidance (Davidson, 1998). In the present study, the non-anxious depressed group showed a reduced degree of defensive action during a nociceptive threat (i.e., hand withdrawal), which was preserved in anxious depression. Future research should examine how those with anxious vs. non-anxious depression respond to treatments targeting the defensive action system. Compared to a group with anxiety only, it seems possible that an anxious depressed group might show both reduced approach and increased avoidance (e.g., using approach/avoidance threat paradigms). Further studies are needed to tease apart the potentially unique disease that the intersection of anxiety and depression represents.

We previously proposed (Ironside et al., 2022) a process model for anxiety and anxious depression to help distinguish disease modifiable processes that could be useful targets in treatment development. Individuals with anxiety may be characterized by an excessive defense system that takes incoming or internally generated stimuli and evaluates them with respect to threat-to-self, with a high prior expectation of aversive outcomes (Paulus et al., 2019). This process may be highly taxing and may result in exhausting affective processing capacities ultimately resulting in depression. This proposition is consistent with the observation that two-thirds of individuals with lifetime comorbid anxiety disorders and MDD reported an earlier age-of-onset of their first anxiety disorder than their MDD (Kessler et al., 2015). It is unclear whether this subsequent depression is characterized by a lack of response to both positive/negative affect, i.e., if anxious depression often becomes non-anxious depression eventually. It is also unlikely that the entire sub-group will show the same phased process. In comparison, depressed-only individuals might be characterized by a primary lack of reactivity to positive and negative stimuli, which results in a lack of anxious responding even when threatening stimuli are encountered. Therefore, whereas the primary disease-modifying process for anxious depression would be to attenuate threat-related processing, the primary disease-modifying process for non-anxious depression would be to enhance valence-independent motivation in general. A lot of focus is given to reward responsivity in depression, but we propose a more general factor of blunted motivation (both approach and avoidance motivation), supported by our prior (Ironside et al., 2022) and current findings. This would modify the tripartite model, transforming the anxiety-specific factor of hyperarousal into a general “arousal/motivation” factor, with depression and anxiety pulling in opposing directions. One consequence of this suggestion would be that treatments targeting hyperarousal in non-anxious depression could actually worsen an already blunted emotional reactivity. Interestingly, the same pattern of blunted aversive interoception was found when comparing the larger T500 study sample in terms of their past suicide attempt history, with the past attempters showing higher pain tolerance in the cold pressor challenge and less suffocation on a breath-hold task (DeVille et al., 2020, 2021). Perhaps this is descriptive of the process model suggested above, with initial anxious depression as a predecessor to the potentially more chronic state of non-anxious depression. However, this is at odds with worse reported outcomes and higher rates of suicidality in anxious depression.

The present study had several limitations. First, case-control designs and cross-sectional studies cannot support causal inferences. Longitudinal designs will be necessary to determine cause and effect. Second, the Dep+Anx group was twice as large, which may have increased the statistical power to detect effects in the Dep+Anx vs. the Dep-only group. However, as there were no group differences in the heartbeat task or the VIA tasks, this concern does not appear to have indiscriminately influenced the current findings. Third, we used a somewhat imprecise, albeit commonly employed, measure of nociceptive stimulation in the cold-pressor challenge. It would be advantageous to clarify whether anxious depression is associated with impaired processing of other pain signals, such as heat (Thompson et al., 2008) or pinprick forms of pain. Furthermore, heartbeat perception tasks have been the subject of criticism, reflecting the difficulty of assessing cardiac interoception at rest (Khalsa and Lapidus, 2016). We attempted to overcome this with our endogenous perturbation approach (i.e., breath-hold incorporation), but other exogenous perturbation approaches (e.g., Teed et al., 2022) are worth utilizing in future investigations. In addition, according to our power calculations our fMRI analyses were underpowered and therefore could only be deemed exploratory. Finally, without including an anxiety-disorder only comparator group it is unclear if the increased reactivity observed in our sample is due to anxiety, or whether the interaction of depression and anxiety has a unique profile. Future studies should assess differences in interoceptive and nociceptive processing between those with anxiety disorders only, depression only, and comorbid anxiety and depression. We did not find significant differences between the groups on a number of measures. However, those that involve aversive sensations such as the Cold-Pressor Challenge and the Breath-Hold Challenge showed group differences. This could be because of the limitations of the tasks outlined above, or, it could be indicative of the group differences stemming from the processing of aversive sensations only and thus driven by differences in threat sensitivity. Additionally, the fact that there were no statistically significant group differences in the activity of insula subregions during interoceptive attention (possibly due to low statistical power) limits our ability to dwell further on the possible neural correlates of differences in aversive interoceptive reactivity in individuals with anxious depression vs. those with depression alone. One possibility is that the observed differences in perception are related to activity in brain regions other than the insular cortex, although further study would be needed to verify this notion.

## Conclusion

In sum, these findings provide further support for the view that anxious and non-anxious depression have distinct neurocognitive and neurophysiological profiles. One outcome of this arrangement may be that exaggerated reactivity to aversive interoceptive sensations in anxious depression could promote avoidance behavior, whereas a lack of valence-independent motivation could be a driving factor of similar outcomes in non-anxious depression. Our findings add to the growing body of evidence that interoceptive and nociceptive signals are processed differently in individuals with comorbid depression and anxiety than in individuals with depression alone, providing support for a process model of increased threat sensitivity and hyperarousal in anxious depression.

## Data availability statement

The raw data supporting the conclusions of this article will be made available by the authors pending the establishment of data sharing agreements between all parties.

## Ethics statement

The studies involving human participants were reviewed and approved by Western Institutional Review Board. The participants provided their written informed consent to participate in this study prior to study entry, and received compensation for their study involvement.

## Author contributions

MP and SK: study conception and design. MI, DD, RK, AT, KB, RS, MP, and SK: analysis and interpretation of results. MI, DD, and SK: draft manuscript preparation. All authors contributed to the article and approved the submitted version.
