# Supplementary material for: The unique face of comorbid anxiety and depression: increased interoceptive fearfulness and reactivity
Source: Front Behav Neurosci. 2023 Jan 23;16:1083357. doi: 10.3389/fnbeh.2022.1083357 (PMC9899910; doi:10.3389/fnbeh.2022.1083357)
Supplement: Supplementary file 1 [file Data_Sheet_1.docx]

**SUPPLEMENTAL INFORMATION**

**Supplemental Methods:**

**Parent Study Methods:**

The following supplemental materials summarizes the methods from the parent study. Full details are in the protocol paper ([Victor et al., 2018](#_ENREF_4)).

**Parent Study Participants:** We collected datasets on a total of 1050 participants with approximately 506 mood and/or anxiety, 330 substance use, 54 eating disorder and 160 mentally and physically healthy control participants. In order to obtain at least 1000 participants who completed the year-long study, we enrolled 1272 participants between January 2015 and December 2018. Participants were between 18 and 55 years of age and had a body mass index between 17-38kg/m^2^. Participants were either referred from local treatment facilities or seeking treatment for anxiety and/or depressive symptoms, problems related to substance use, or problems related to eating behavior. As part of the inclusion criteria, mood/anxiety, substance, and eating disorder participants must have also screened positive for these conditions as indicated by a score on the Patient Health Questionnaire (PHQ-9) ≥ 10 and/or Overall Anxiety Severity and Impairment Scale (OASIS) ≥ 8, (DAST-10) score > 2 or Sick, Control, One, Fat, Food Questionnaire eating disorder screen (SCOFF) score ≥ 2. Participants who met criteria for one primary domain could also screen positive for one of the other study domains. Healthy control participants screened negative for these inclusion measures.

**Parent Study Design:**The study’s dependent variables focus on the *positive and negative valence systems, cognition, and arousal/interoception domains* proposed by the RDoC ([Health, 2011b](#_ENREF_2), [Health, 2011a](#_ENREF_1)). Using self-report, behavior, physiology, neural circuit, cell, molecule, and gene unit of analysis measures, these constructs were applied to a clinical population of individuals with dysregulation of affect, substance use, and eating behavior recruited from treatment providers across different sites in the community. Participants underwent a multi-level assessment based on the RDoC approach that consists of (a) a standardized diagnostic assessment, (b) self-report questionnaires assessing the positive and negative valence domains as well as interoception, (c) behavioral tasks assessing positive and negative valence, cognition, and interoception, (d) physiological measurements consisting of skin conductance, facial emotion expression monitoring, heart rate, respiration and eye-blink startle response, (e) functional magnetic resonance imaging focusing on reward-related processing, fear conditioning and extinction, cognitive control and inhibition, and interoceptive processing, (f) biomarker assessment, (g) microbiome assessment, (h) blood to derive induced pluripotent stem cells (IPS), (i) and genetic as well as epigenetic assessments. Subsequently, these individuals were followed up quarterly and for one year. At months 3, 6, and 9, only self-report assessments were collected, and the participants were re-assessed using a multi-domain assessment of functioning, which included: (a) symptom severity and duration, (b) subjective well-being, (c) psychosocial function, (c) occupational function, (d) physical health, (e) utilization of mental health resources (treatment), and (f) adherence to treatment.

The workflow schematic in Figure S1 describes the overall outline of the T-1000 study and the measures obtained at different points in time.


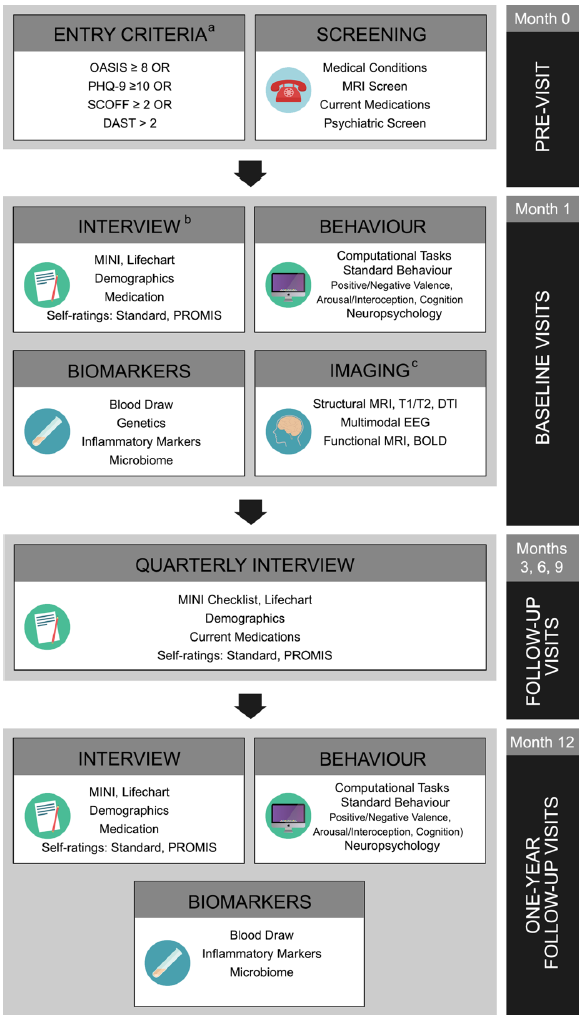


**Supplemental Figure S1: Tulsa 1000 workflow schematic.** BOLD, blood oxygen level-dependent; DAST, drug abuse screening test; DTI, diffusion tensor imaging; EEG, electroencephalogram; MINI, Mini International Neuropsychiatric Interview; OASIS, Overall Anxiety Severity and Impairment Scale; PHQ-9, Patient Health Questionnaire; PROMIS, Patient-Reported Outcome Measurement Information System; SCOFF, Sick,Control, One, Fat, Food Questionnaire; T1/T2, T1-weighted (longitudinal relaxation time) and T2-weighted (transverse relaxation time). Reproduced with permission from ([Victor et al., 2018](#_ENREF_4)).

**Beat-to-tap consistency measure:**

For a full description of the measure see ([Smith et al., 2021](#_ENREF_3)). Briefly, the beat-to-tap consistency measure was calculated by first assigning each response to the nearest heartbeat (or tone) and calculating the time difference between the two—such that each response was assigned a response time, which could be positive or negative. The decision to use the nearest heartbeat (or tone) event, rather than the previous event was based on the observation that some participants tended to respond preemptively to repeated salient events. First, we calculated the standard deviation of the response times. This measure, however, was expected to be correlated with actual heart rate, due to the fact that faster heart rates yield shorter windows during which a participant may respond, so that a participant tapping randomly will tend to appear more precise. We estimated the distribution of expected values under random tapping using a participant’s actual recorded heart beats and actual number of responses, placed randomly using a uniform distribution for the minute trial. We then converted a participant’s actual tapping behavior into a Z-score by subtracting the mean and dividing by the standard deviation of the estimated distribution (see Fig. S2). In this way, we calculated a perceptual measure that was corrected for, and no longer correlated with, actual heart rate: the beat-to-tap consistency. Note that, as a result, the beat-to-tap consistency measure also does not depend on individual differences in reaction times (e.g., as would be a concern if one instead simply measured the average temporal distance between heartbeats/tones and taps).


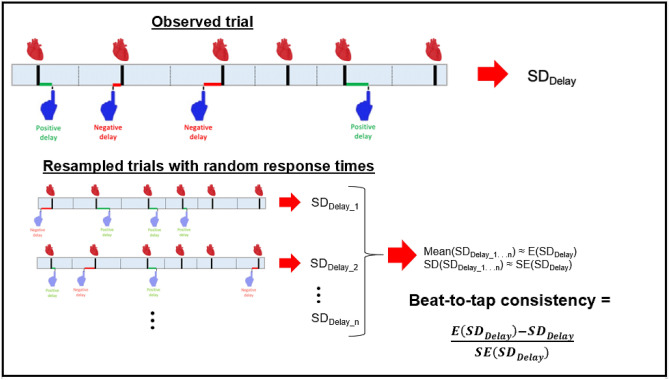


**Supplemental Figure S2: Beat-to-tap consistency measure.** Illustration of how beat-to-tap consistency was calculated as a measure of reliability in the temporal relationship between participants’ heartbeats (shown as thick black lines with heart images above them) and taps (shown as blue hands, with either positive or negative temporal distances from each heartbeat) that was uncorrelated with their heart rate. This involved first estimating the distribution of expected (standard deviation; SD) values under random tapping using a participant’s actual recorded heartbeats and actual number of responses, placed randomly (indicated below by partially transparent hand images) using a uniform distribution for the trial over large numbers of simulated trials (here n = 1000 trials). Then a participant’s actual tapping behavior was converted into a Z-score by subtracting it from the mean and dividing by the standard deviation of the estimated distribution. Reproduced with permission from ([Smith et al., 2021](#_ENREF_3)).

**Supplemental References:**

HEALTH, N. I. O. M. 2011a. *Negative Valence Systems: Workshop Proceedings* [Online]. Rockville, MD: NIMH. Available: <http://www.nimh.nih.gov/research-funding/rdoc/negative-valence-systems-workshop-proceedings.shtml> [Accessed 10/12/2012 2012].

HEALTH, N. I. O. M. 2011b. *Positive Valence Systems: Workshop Proceedings* [Online]. Rockville, MD: NIMH. Available: <http://www.nimh.nih.gov/research-funding/rdoc/positive-valence-systems-workshop-proceedings.shtml> [Accessed 10/12/2012 2012].

SMITH, R., FEINSTEIN, J. S., KUPLICKI, R., FORTHMAN, K. L., STEWART, J. L., PAULUS, M. P., TULSA, I. & KHALSA, S. S. 2021. Perceptual insensitivity to the modulation of interoceptive signals in depression, anxiety, and substance use disorders. *Scientific reports,* 11**,** 2108-2108.

VICTOR, T. A., KHALSA, S. S., SIMMONS, W. K., FEINSTEIN, J. S., SAVITZ, J., AUPPERLE, R. L., YEH, H. W., BODURKA, J. & PAULUS, M. P. 2018. Tulsa 1000: a naturalistic study protocol for multilevel assessment and outcome prediction in a large psychiatric sample. *BMJ Open,* 8**,** e016620.
